# Elicitation of Potent H5N1- and H7N9-Neutralizing Antibody Responses in Rhesus Macaques by Sequential Heterologous Vaccination with mRNA and Adenoviral Vectors Encoding Full-Length Hemagglutinins

**DOI:** 10.3390/v18090981

**Published:** 2026-09-07

**Authors:** Brandon C. Rosen, Jack T. Mauter, Thomas B. Voigt, Giovana de Figueiredo Godoy, Emma L. Walker, Johan J. Louw, Noor Ghosh, Christakis Panayiotou, Aaron Yrizarry-Medina, Joshua Terao, Eva G. Rakasz, Matthew R. Reynolds, Dawn M. Dudley, Douglas S. Reed, David I. Watkins, Michael J. Ricciardi

**Affiliations:** 1 Department of Pathology, George Washington University School of Medicine and Health Sciences, Washington, DC 20052, USAmricciardi@gwu.edu (M.J.R.); 2 Medical Scientist Training Program, University of Miami Miller School of Medicine, Miami, FL 33136, USA; 3 Center for Vaccine Research, University of Pittsburgh, Pittsburgh, PA 15261, USA; 4 Wisconsin National Primate Research Center, University of Wisconsin-Madison, Madison, WI 53715, USA; 5 Mabloc, LLC, Washington, DC 20052, USA

**Keywords:** highly pathogenic avian influenza, H5N1, H7N9, vaccines, antibodies, B lymphocytes, influenza A virus

## Abstract

Highly pathogenic avian influenza (HPAI) represents an enormous pandemic risk amid the ongoing H5N1 panzootic. HPAI, defined by its high lethality in domestic poultry, includes the influenza A virus (IAV) H5N1 and H7N9 subtypes and has a 40–50% case fatality rate in humans. To facilitate the development of efficacious HPAI vaccination regimens and isolation of HPAI-neutralizing monoclonal antibodies (nmAbs) for prophylactic and therapeutic use, we performed a proof-of-concept pilot study wherein we developed an array of mRNA and adenovirus-vectored vaccines encoding HPAI hemagglutinins (HAs) and then assessed their immunogenicity in IAV-naïve Indian rhesus macaques (RMs). All RMs developed binding IgG recognizing both HPAI HAs. HPAI-nAbs were detected in RMs vaccinated with full-length HAs, but not in RMs vaccinated with HA stems alone. Potent HPAI neutralization activity was observed in two animals with serum ID_50_ titers of ~1:100,000 against H5N1 and ~1:50,000 against H7N9. Most RMs developed cross-reactive IgG recognizing the HAs of additional IAV subtypes, and HA-specific B cells were readily identifiable in vaccinee PBMCs by flow cytometric analysis. The results of our pilot study suggest that elicitation of potent HPAI-nAb responses is enhanced by vaccination with the HA head domain and that vaccination with the HA stem alone tends to elicit binding non-nAbs. Furthermore, use of our fluorophore-conjugated HA probes to identify HA-specific B cells could enable HPAI-nmAb isolation. Collectively, our findings could facilitate the development of novel vaccines and nmAb therapeutics leveraging the superior neutralization potency of HA head-specific Abs to prevent and treat HPAI infections in humans.

## 1. Introduction

The highly pathogenic avian influenza (HPAI) H5N1 and H7N9 subtypes pose enormous threats to human health and possess considerable pandemic potential. HPAI is lethal in 40–50% of human infections [1]. During the past five years of the ongoing H5N1 panzootic, high levels of viral replication, proliferation, and dissemination have occurred, resulting in the deaths of hundreds of millions of birds and tens of thousands of mammals [2,3,4,5]. The current H5N1 strains in circulation appear to have greater transmissibility among both avian and mammalian species, as well as an enhanced tropism for mammalian cells, relative to earlier strains [6,7,8,9]. The ongoing panzootic has created numerous opportunities for crossover infections of humans and, indeed, there have been 71 confirmed H5N1 infections in humans in the U.S. as of late 2025 [10]. Although most human cases in the U.S. have been mild and nonlethal during the current outbreaks, this could be the result of inefficient transmission through virus-laden bovine milk splashing into the eyes of farm workers, causing conjunctivitis with minimal respiratory symptoms [11]. Pre-existing immunity to the seasonal *Alphainfluenzavirus influenzae* (influenza A virus; IAV) H1N1 subtype may also provide some degree of protection against lethal H5N1 infection [12,13,14]. H5N1 viruses isolated from dairy cattle and farmers in the ongoing outbreak have been shown to be lethal in animal models [12,15,16]. However, if human inoculation via respiratory droplets or aerosols occurs, mortality rates could increase to the 40–50% historical case fatality rates. It is therefore imperative that effective prophylactic and therapeutic countermeasures for HPAI are rapidly developed and optimized for human use.

Existing vaccines and antiviral therapeutics may provide some efficacy against HPAI in humans but are limited by several factors. Seasonal influenza vaccinations are widely available but only include the H1N1 and H3N2 IAV subtypes (along with one influenza B virus [IBV] subtype), resulting in antibody (Ab) responses that react poorly with HPAI [17,18,19,20]. Several H5N1 vaccines have been approved for use in humans and elicit H5N1-neutralizing Abs (nAbs) [21,22,23]. However, these vaccines are formulated with H5N1 strains that are 20 years old, which could reduce the potency of vaccine-induced nAbs effective against the current H5N1 strains in circulation. Additionally, H5N1 vaccines likely do not elicit Ab responses that potently cross-neutralize H7N9, meaning that individuals would need a separate H7N9-based vaccination for protection against both high-lethality HPAI subtypes. Finally, the primary therapeutic agents available for influenza infections in humans—neuraminidase inhibitors—have limited efficacy, are susceptible to viral resistance mutations, and have undesirable and sometimes dangerous side-effect profiles [24,25,26,27,28,29,30,31,32,33,34].

To enhance preparedness for possible HPAI epidemics and pandemics, we performed a novel HPAI-directed vaccination study in nonhuman primates (NHPs) to facilitate the future development of human HPAI vaccines and neutralizing monoclonal Ab (nmAb) therapeutics. We aimed to provide coverage against both highly lethal HPAI subtypes and elicit nmAbs that cross-react with both HPAI subtypes as well as the seasonal IAV subtypes. We designed novel recombinant HPAI HA proteins and HA subdomains for expression from mRNA-lipid nanoparticles (mRNA-LNPs) and recombinant adenovirus type 5 (rAd5) vectors, then administered these HPAI HA-encoding vaccines to six Indian rhesus macaques (RMs; *Macaca mulatta*) in three distinct heterologous prime–boost–boost vaccination regimens. To maximize the likelihood of eliciting both potent and broadly reactive nAb responses, our three vaccination regimens comprised (1) vaccines encoding the H5N1/H7N9 HA stem regions only to elicit broadly reactive Ab responses, (2) vaccines encoding the full-length H5N1/H7N9 HAs only to elicit potent Ab responses, and (3) vaccines encoding H5N1-derived HAs (full-length and stem) only to elicit potent H5N1-nAb responses, since H5N1 poses the most imminent threat to humans amid the ongoing panzootic. Importantly, in contrast to humans—who are exposed to a number of different influenza strains throughout their lives through vaccination and natural infection—our RM vaccinees showed no serological evidence of prior IAV exposures, thereby permitting the analysis of HPAI-specific immune responses in the absence of prior exposure.

Herein, we report the immunogenicity and efficacy of our vaccination regimens in eliciting nAb responses against both HPAI subtypes as well as the seasonal IAV subtypes. Additionally, we demonstrate that HA-specific B cells can be readily identified in our vaccinees by flow cytometric analysis, thereby facilitating the isolation of mAbs for potential development as HPAI therapeutic agents. Finally, we discuss the implications of our findings in the contexts of influenza vaccine development and further efforts to develop HPAI-specific nmAbs for both prophylactic and therapeutic use.

## 2. Results

### 2.1. HPAI HAs Are Efficiently Expressed from mRNA-LNPs and rAd5 Vectors In Vitro

**Figure 1:**
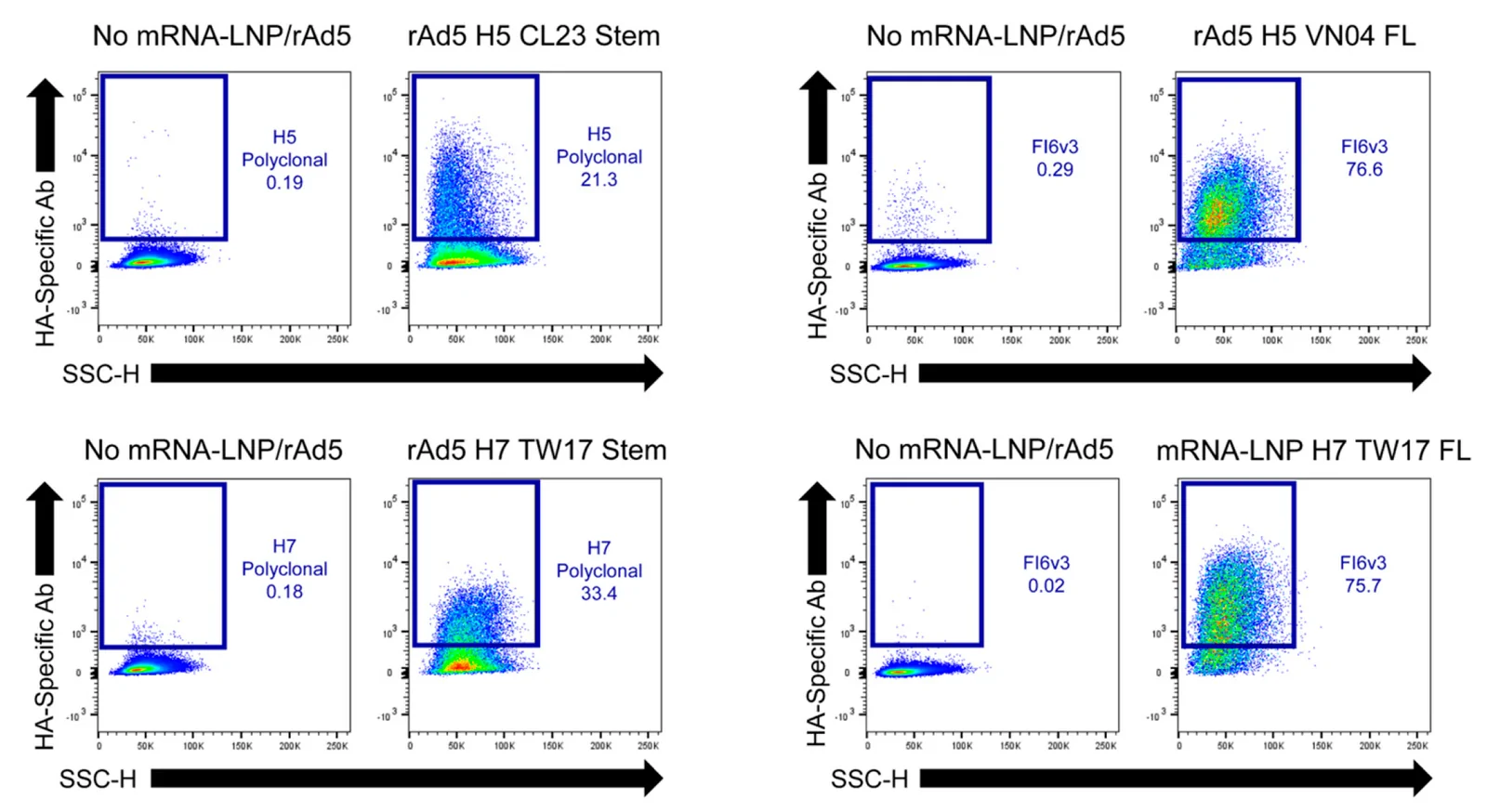
In vitro validation of rAd5- and mRNA-LNP-based HPAI HA vaccines. Expi293F cells were transduced with the indicated rAd5 vectors or transfected with the indicated mRNA-LNPs. Surface HA expression was assessed by staining cells with the indicated HA-specific Abs and a fluorophore-conjugated anti-IgG secondary Ab at 48 h post-transduction/transfection, then analyzing cells by flow cytometry. Plots depict all live cells from representative experiments.

To elicit broadly reactive Ab responses capable of neutralizing both the H5N1 and H7N9 HPAI subtypes, we designed multiple vaccination constructs encoding the full-length HAs and membrane-bound HA stem domains of several HPAI strains. We first assessed the expression of our putative vaccination constructs via DNA transfection (pcDNA3.4 backbone) of Expi293F cells, followed by flow cytometric analysis at 48 h post-transfection using primary HA-specific mAbs and secondary fluorophore-conjugated anti-IgG Abs for staining. Vaccination constructs exhibiting the best expression from pcDNA3.4 were selected for mRNA-LNP and rAd5 vector production. We then confirmed proper surface HA expression from our mRNA-LNPs and rAd5 vectors via transfection and transduction of Expi293F cells, respectively, followed by the flow cytometric analysis described above. Vaccines encoding full-length HAs exhibited high-level surface expression from both mRNA-LNPs and rAd5 vectors, while vaccines encoding membrane-bound HA stems exhibited poorer expression (Figure 1).

### 2.2. HPAI HA Vaccination Elicits H5N1- and H7N9-Binding Abs in RMs

We selected six RMs from our previous SIV vaccination and challenge studies for inclusion in our HPAI vaccination study (Table 1). Two RMs were previously vaccinated against SIV but were never infected, while the other four RMs were infected with SIVmac239 but were all Mamu-B*08+ elite controllers, maintaining low viral loads (chronic-phase viremia ≤10,000 vRNA copies/mL plasma) and exhibiting no opportunistic infections indicative of SIV-induced disease progression to AIDS for approximately two years prior to the present study [35,36,37]. All six animals were IAV-naïve based on HA ELISAs showing undetectable serum binding titers against HA trimers corresponding to the H1, H3, H5, and H7 IAV subtypes.

**Figure 2:**
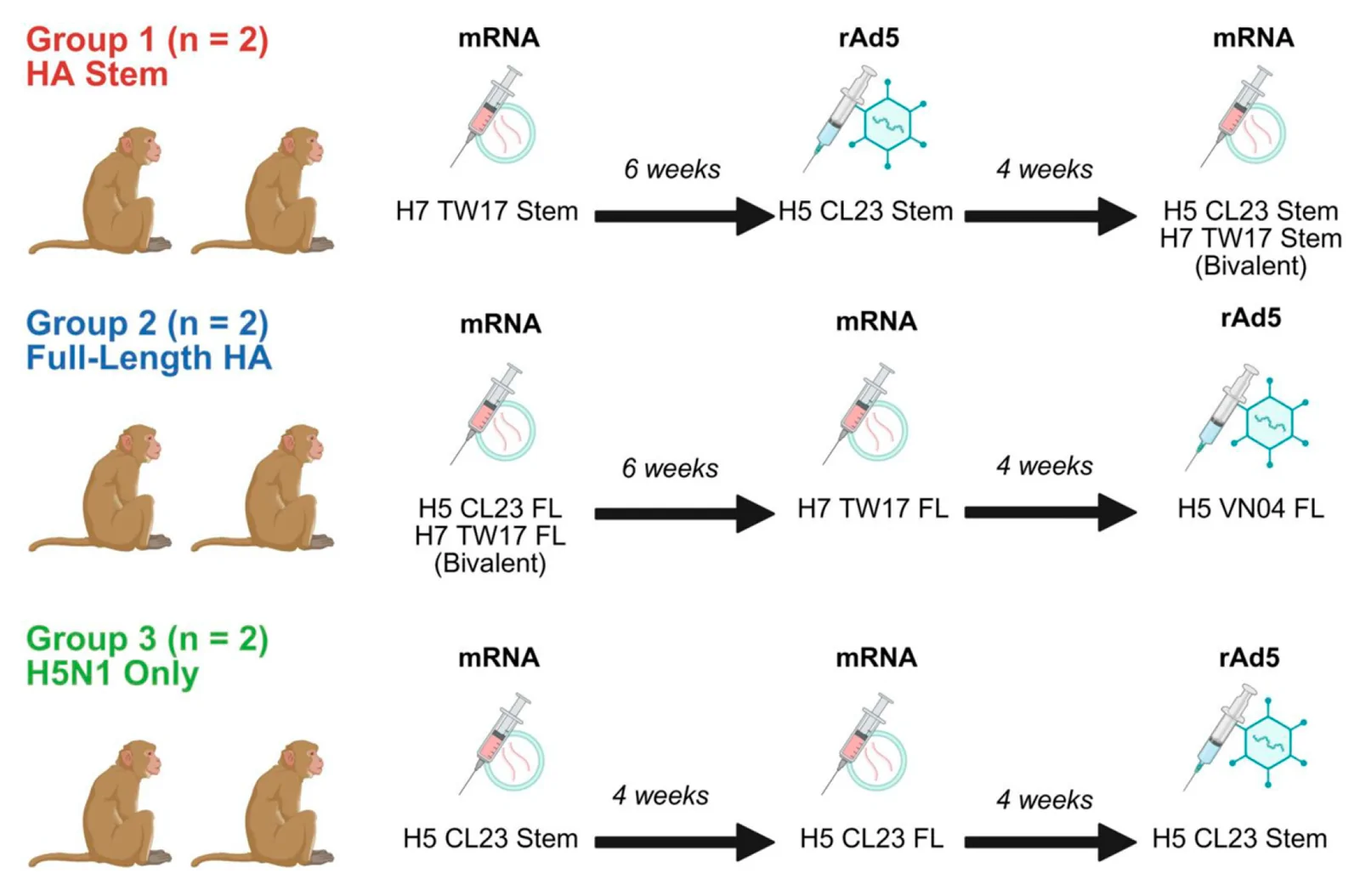
HPAI vaccination study design. Six IAV-naïve RMs were divided into three vaccination groups. Each group of RMs received three intramuscular vaccinations, spaced 4–6 weeks apart, with rAd5 vectors and/or mRNA-LNPs encoding the indicated full-length HAs (FL) or membrane-bound HA stems (STM). Graphic was created using BioRender.com.

These six RMs were divided into three groups of two animals, each receiving a unique three-dose vaccination regimen aimed at eliciting broadly reactive and/or potent HPAI-neutralizing Abs (Figure 2). Group 1 was vaccinated with mRNA-LNPs and an rAd5 vector encoding membrane-bound HA stems of H5N1 (A/Chile/25945/2023 [CL23]) and H7N9 (A/Taiwan/1/2017 [TW17]) to elicit Abs exhibiting the broadest IAV HA reactivity. Group 2 was vaccinated with mRNA-LNPs encoding the full-length HAs of H5N1 CL23 and H7N9 TW17 and an rAd5 vector encoding the full-length HA of H5N1 (A/Vietnam/1203/2004 [VN04]), with the objective of eliciting both cross-reactive Abs but also higher overall HA-specific Ab titers due to the superior immunogenicity of the HA head region. Since H5N1 poses the most imminent human epidemic and pandemic threat, Group 3 was vaccinated with mRNA-LNPs encoding the full-length HA and membrane-bound HA stem of H5N1 CL23 and an rAd5 vector encoding the membrane-bound HA stem of H5N1 CL23 to elicit high titers of H5N1-nAbs.

**Figure 3:**
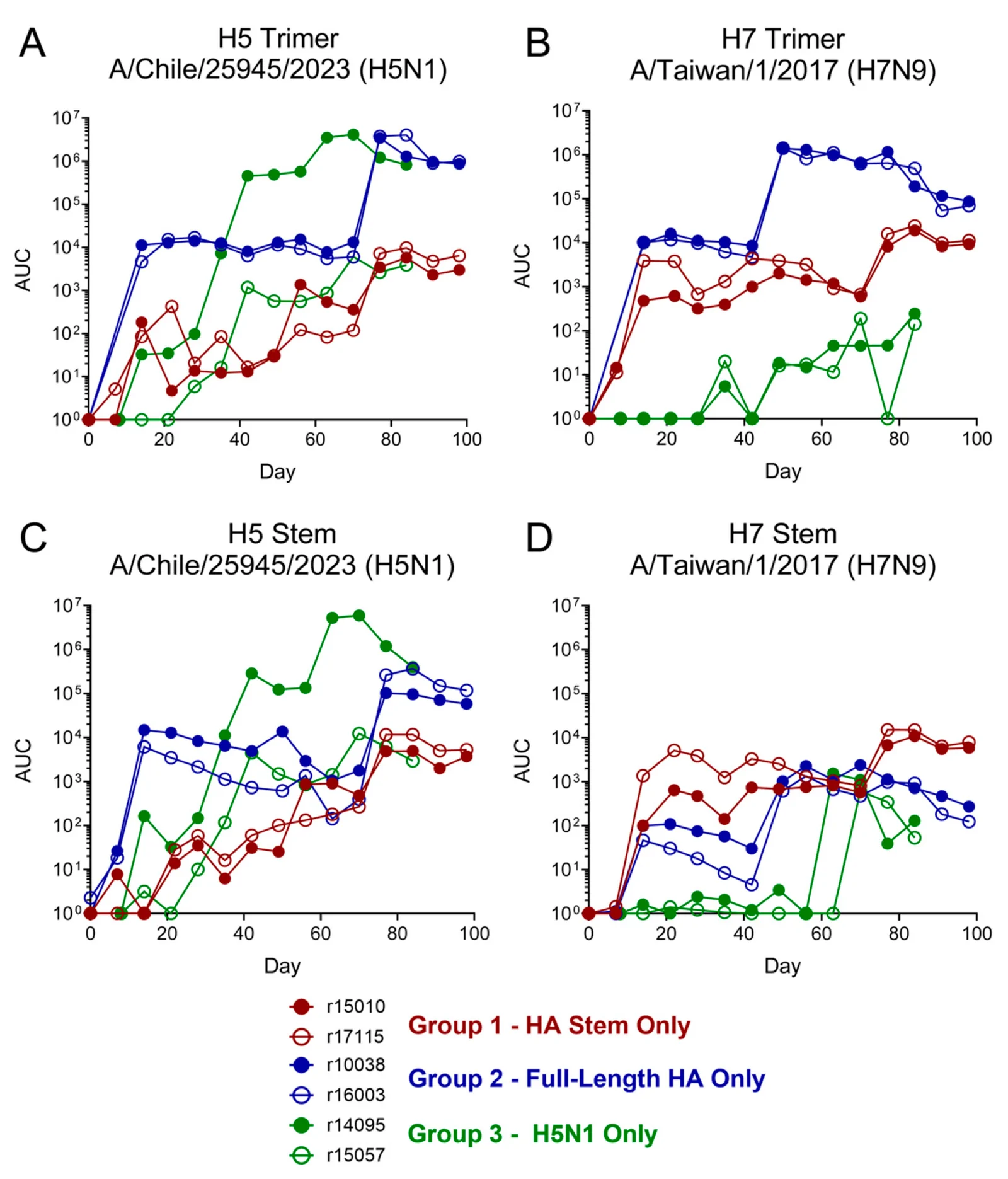
Longitudinal titers of HPAI HA trimer- and stem-binding IgG in vaccinated RMs. Serum HPAI HA binding activity was evaluated by ELISA using recombinant (**A**) H5N1 HA trimer, (**B**) H7N9 HA trimer, (**C**) H5N1 HA stem, and (**D**) H7N9 HA stem. Plots depict levels of IgG binding the indicated recombinant HA protein, represented as area under the curve (AUC). Vaccinees received intramuscular injections of mRNA-LNPs and/or rAd5 vectors encoding HPAI HA stems only (Group 1, red), full-length HPAI HAs only (Group 2, blue), or H5N1 HAs only (Group 3, green).

We initially assessed vaccine immunogenicity by HPAI HA trimer and stem IgG ELISAs using vaccinee serum from several timepoints throughout the vaccination regimen. Based on the kinetics of increases in titers of HA-binding IgG, vaccines encoding full-length HAs were more immunogenic than those encoding membrane-bound HA stems (Figure 3). Animals vaccinated with full-length HAs only (Group 2) exhibited the highest titers of IgG binding the H5 and H7 trimers and the H5 stem. Interestingly, titers of H7 stem-binding IgG were substantially lower than titers of H5 stem-binding IgG in all groups, despite high levels of H7 trimer-binding IgG in Group 2 animals. Animals vaccinated against H5N1 only (Group 3) exhibited relatively high titers of binding IgG specific for the H5 trimer and H5 stem, but displayed poor cross-reactivity with the H7N9 HAs. Of note, one Group 3 animal, r15057, exhibited attenuated vaccine-induced Ab responses, likely resulting from SIV-induced immunodeficiency following loss of control of viral replication early in the vaccination regimen. Unsurprisingly, this animal consistently exhibited the poorest vaccine-induced Ab responses in most of our subsequent immunoassays.

### 2.3. HPAI HA Vaccination Elicits Binding Abs That Cross-React with the HAs of Seasonal Influenza and LPAI Subtypes

**Figure 4:**
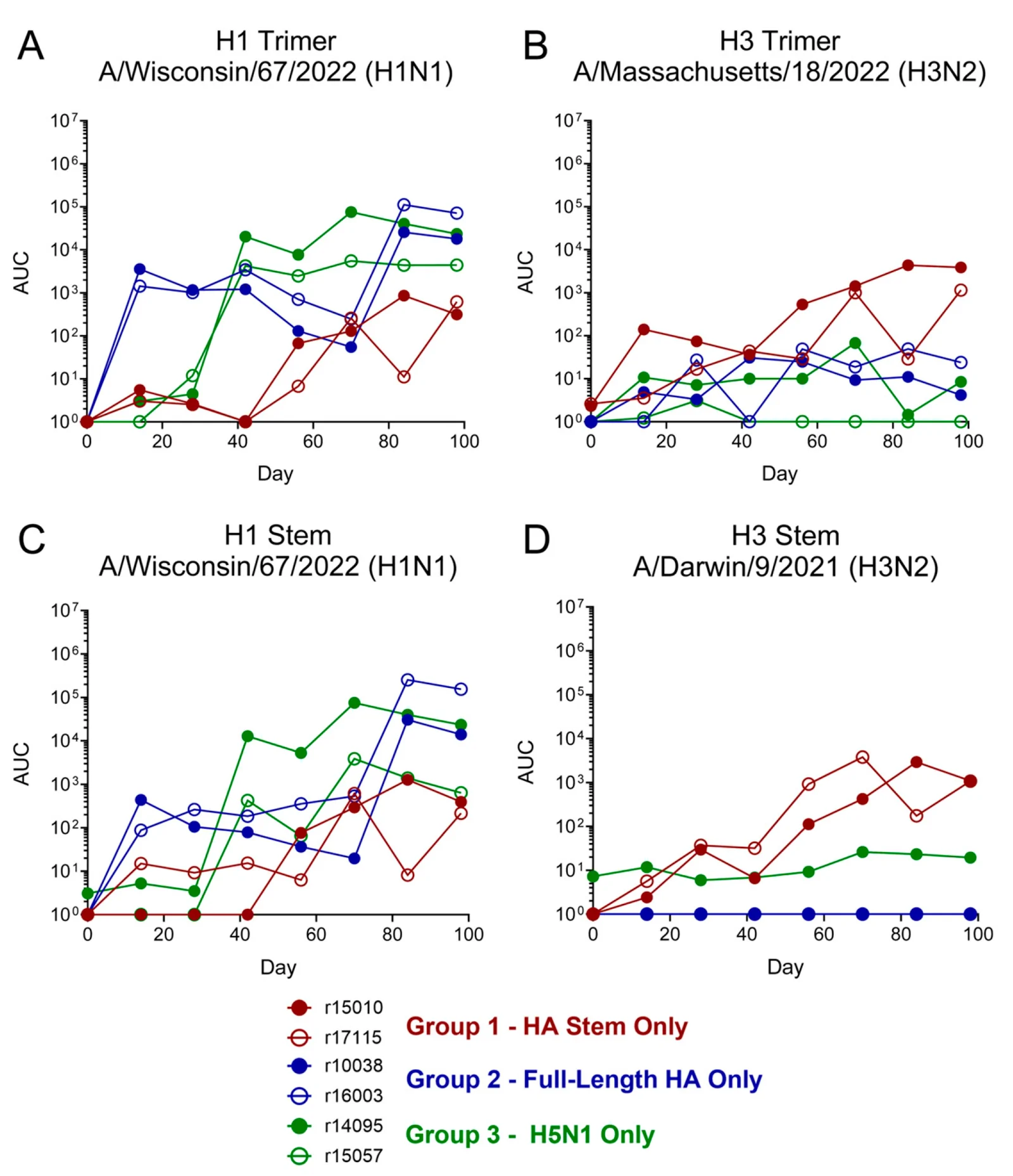
Longitudinal titers of seasonal IAV HA trimer- and stem-binding IgG in vaccinated RMs. Serum seasonal IAV HA binding activity was evaluated by ELISA using recombinant (**A**) H1N1 HA trimer, (**B**) H3N2 HA trimer, (**C**) H1N1 HA stem, and (**D**) H3N2 HA stem. Plots depict levels of IgG binding the indicated recombinant HA protein, represented as area under the curve (AUC). Vaccinees received intramuscular injections of mRNA-LNPs and/or rAd5 vectors encoding HPAI HA stems only (Group 1, red), full-length HPAI HAs only (Group 2, blue), or H5N1 HAs only (Group 3, green).

**Figure 5:**
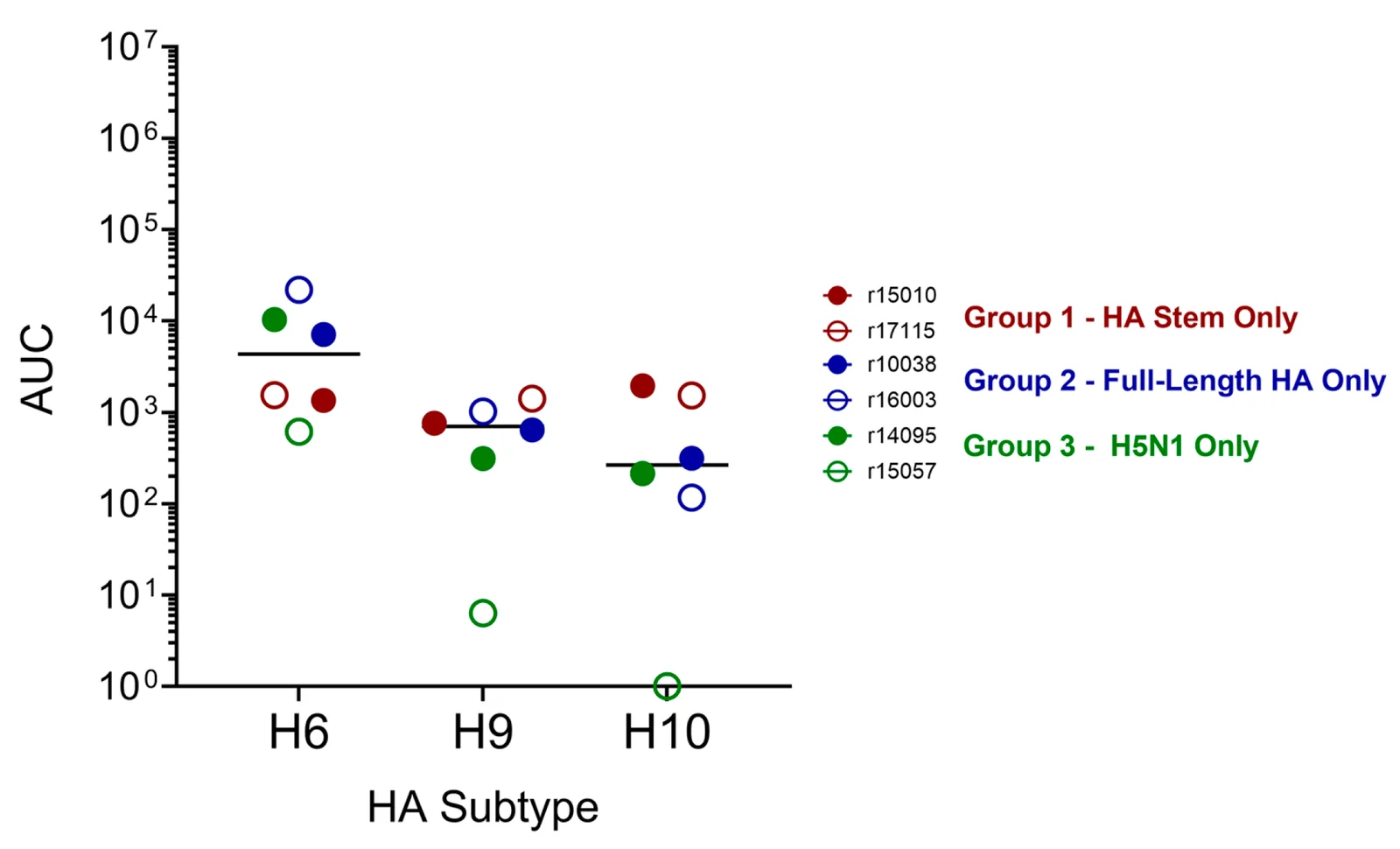
LPAI HA-binding IgG in serum of vaccinated RMs following three vaccine doses. Vaccinee serum from day 98 (four or six weeks after the third vaccination dose, depending on vaccination group) was assayed for LPAI HA monomer-binding IgG by ELISA. Graph depicts levels of IgG binding the indicated recombinant HA protein, represented as area under the curve (AUC). Vaccinees received intramuscular injections of mRNA-LNPs and/or rAd5 vectors encoding HPAI HA stems only (Group 1, red), full-length HPAI HAs only (Group 2, blue), or H5N1 HAs only (Group 3, green). HA monomers correspond to the HA proteins of the following LPAI strains: A/chicken/Guangdong/C273/2011 (H6N2), A/Hong Kong/35820/2011 (H9N2), A/Jiangxi-Donghu/346/2013 (H10N8).

To evaluate the breadth of Ab responses elicited by our HPAI-based vaccination regimen, we assessed longitudinal titers of binding IgG specific for HA trimers and stems corresponding to the seasonal influenza H1N1 and H3N2 subtypes. Relatively high titers of H1 trimer- and stem-binding IgG were detected in Group 2 and Group 3 vaccinees as early as day 14 following the first vaccination dose (Figure 4). The Group 1 animals vaccinated with HA stem alone also mounted H1-binding IgG responses, although titers were lower than those observed in animals vaccinated in regimens containing full-length HA. Interestingly, titers of H3 trimer- and stem-binding IgG were highest in Group 1 animals, while Group 2 and Group 3 animals exhibited near-undetectable H3-binding activity. Next, we assessed whether our overall vaccination regimens elicited binding IgG that cross-react with the H6N2, H9N2, and H10N8 low pathogenicity avian influenza (LPAI) subtypes by comparing serum binding activity at a pre-vaccination timepoint and a final timepoint after all three vaccination doses (98 days after the first vaccination dose). H6N2 HA-binding IgG was detected in the sera of all six vaccinees, while binding IgG recognizing the HAs of H9N2 and H10N8 was detected in the sera of five vaccinees (Figure 5).

### 2.4. Vaccination with Full-Length HAs, but Not Membrane-Bound HA Stems, Elicits H5N1- and H7N9-nAbs in RMs

**Figure 6:**
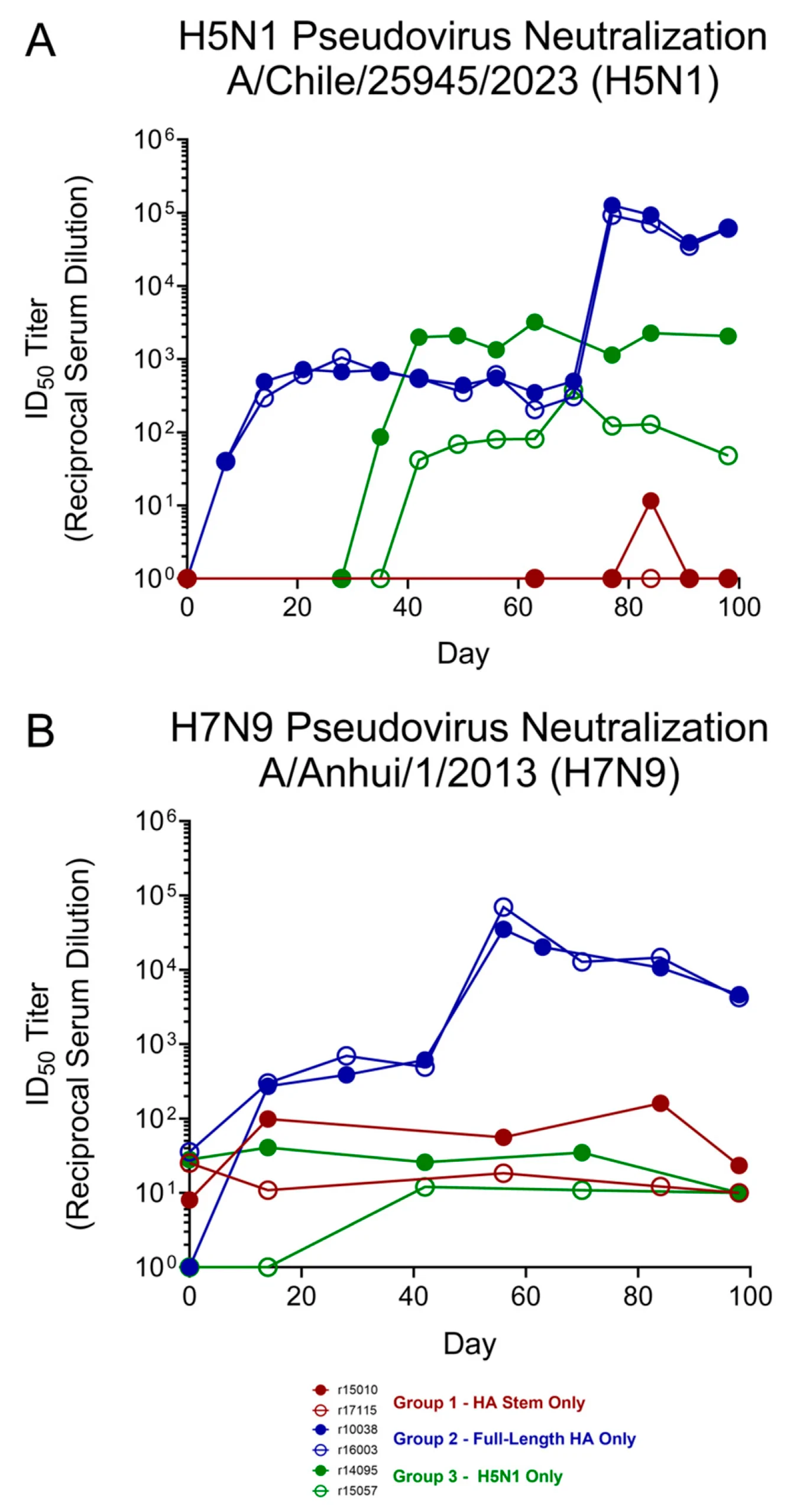
Longitudinal titers of H5N1 and H7N9 PSV-neutralizing Abs in vaccinated RMs. Vaccinee serum neutralization activity against (**A**) H5N1 CL23 (A/Chile/25945/2023) and (**B**) H7N9 AN13 (A/Anhui/1/2013) PSVs. Vaccinees received intramuscular injections of mRNA-LNPs and/or rAd5 vectors encoding HPAI HA stems only (Group 1, red), full-length HPAI HAs only (Group 2, blue), or H5N1 HAs only (Group 3, green). Half-maximal inhibitory dilution (ID_50_) value is defined as the serum dilution reducing PSV infection of MDCKs or MDCK-SIAT1s by 50% relative to the negative control condition lacking rhesus serum.

Next, we assessed the capacity of vaccinee serum to neutralize the H5N1 and H7N9 HPAI subtypes. To first screen our serum samples for HPAI neutralization activity in a BSL-2 setting, we generated lentiviral PSVs composed of an Env- and Vpr-deficient HIV-1 backbone encoding nanoluciferase and full-length HA and NA proteins in the viral envelope. RMs vaccinated with full-length HPAI HA proteins only (Group 2) exhibited the highest titers of H5N1-nAbs, peaking at a half-maximal inhibitory dilution (ID_50_) value near 1:100,000 following the third vaccination with the full-length H5 VN04-encoding rAd5 vector (Figure 6). Animals vaccinated against H5N1 only (Group 3), but with both full-length H5 and membrane-bound H5 stem, began exhibiting H5N1 neutralization activity after the second vaccination dose, but peak ID_50_ values were considerably lower than those of Group 2 (1:3203 for r14095 and 1:376 for r15057). Remarkably, despite possessing moderate titers of H5- and H7-binding Abs, animals vaccinated with membrane-bound HA stems only (Group 1) showed almost no detectable serum H5N1 neutralization activity. To determine whether the neutralization activity assessments obtained using our HPAI PSV system reflected neutralization activity against live influenza viruses, we performed plaque-reduction neutralization tests (PRNTs) with vaccinee serum at selected timepoints using a live H5N1 virus (A/dairy cattle/Texas/24008749001/2024). Peak serum ID_50_ titers obtained via PRNT were consistent with the ID_50_ values obtained from our HPAI PSV neutralization assays (Table 2). Although we observed substantial increases in H5N1 ID_50_ titers between the pre- and post-vaccination timepoints tested, certain animals exhibited relatively high levels of baseline neutralization activity at the pre-vaccination timepoint (ID_50_ as high as 1:1024) despite lacking detectable H5N1 HA-binding Abs or H5N1 PSV-nAbs pre-vaccination. These data suggest that complement, which may be elevated even in controlled HIV/SIV infection [38], or sialylated serum proteins may be contributing to this baseline neutralization activity. 

To explore further associations between vaccine-induced HPAI neutralization titers and the HA domain specificity of these neutralizing Abs, we performed correlation analyses between immunological parameters in the five immunocompetent vaccinees (all except r15057) at a timepoint four weeks after the third vaccination dose. Comparisons of serum H5N1 PSV neutralization activity with titers of H5N1 HA trimer- and stem-binding Abs did not yield statistically significant *p*-values. The association between H5N1 neutralization titers and H5N1 HA trimer-binding Ab titers was stronger (Spearman r = 0.8721, *p* = 0.1000) than the association between H5N1 neutralization titers and H5N1 HA stem-binding Ab titers (Spearman r = 0.5643, *p* = 0.4000) (Table 3). In the four vaccinees receiving at least one dose of an H7N9 HA-encoding vaccine (Group 1 and 2 animals), there was a weak positive association between serum H7N9 neutralization titers and H7N9 HA trimer-binding Ab titers (Spearman r = 0.8000, *p* = 0.3333) and a weak negative association between serum H7N9 neutralization titers and H7N9 HA stem-binding Ab titers (Spearman r = −0.8000, *p* = 0.3333).

### 2.5. Vaccine-Induced Abs Cross-Neutralize Seasonal IAV Subtypes H1N1 and H3N2

**Figure 7:**
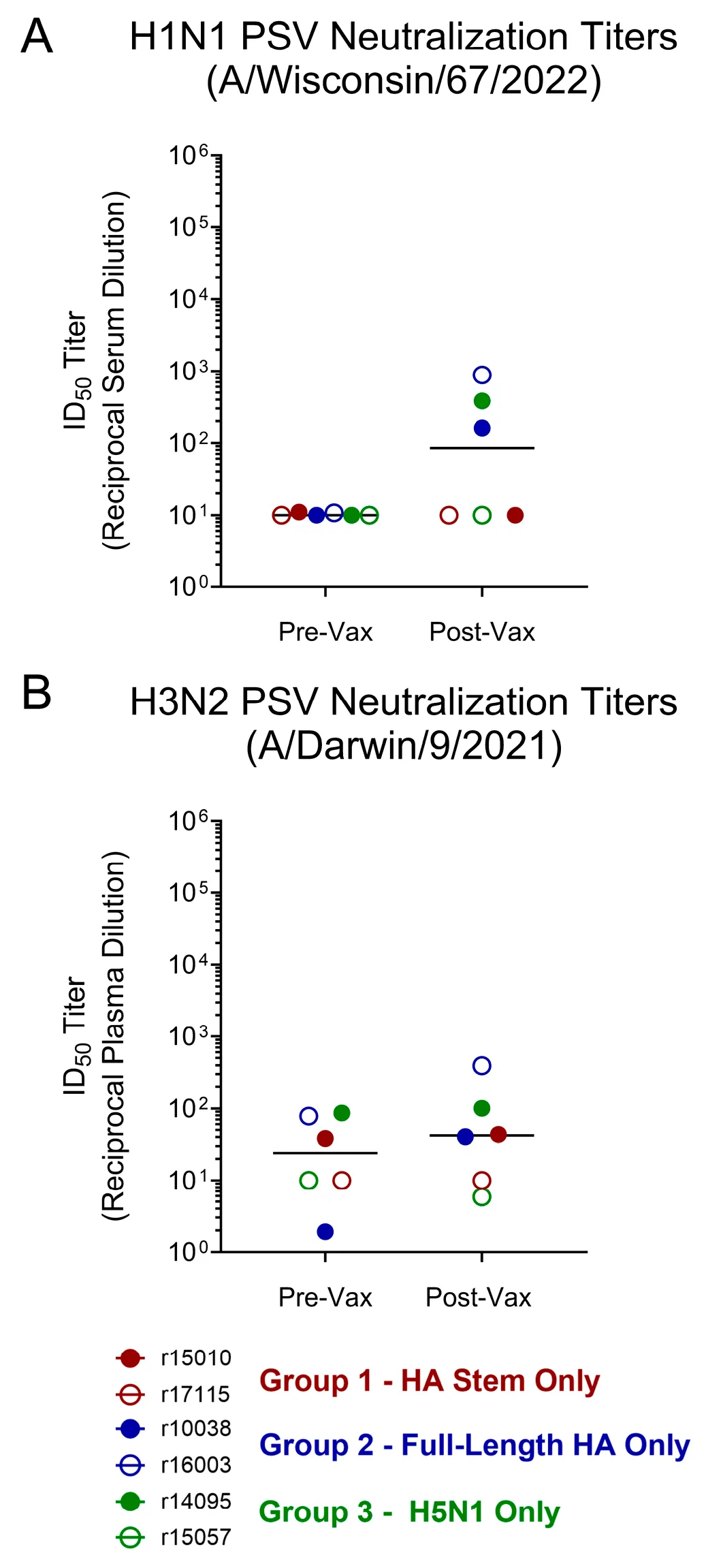
Vaccinee serum/plasma neutralization activity against seasonal IAV subtypes H1N1 and H3N2. (**A**) Vaccinee serum neutralization activity against (**A**) H1N1 WI22 (A/Wisconsin/67/2022) PSV and (**B**) vaccine plasma neutralization activity against H3N2 DW21 (A/Darwin/9/2021) PSV. Vaccinees received intramuscular injections of mRNA-LNPs and/or rAd5 vectors encoding HPAI HA stems only (Group 1, red), full-length HPAI HAs only (Group 2, blue), or H5N1 HAs only (Group 3, green). Half-maximal inhibitory dilution (ID_50_) value is defined as the serum or plasma dilution reducing PSV infection of MDCK-SIAT1s by 50% relative to the negative control condition lacking rhesus serum/plasma.

To more thoroughly assess the breadth of vaccine-induced Ab responses in our RM vaccinees, we performed neutralization assays with IAV PSVs corresponding to the seasonal IAV H1N1 and H3N2 subtypes. Animals receiving at least one dose of a vaccine encoding the full-length H5N1 HA (Groups 2 and 3) developed nAb responses against our H1N1 PSV (pseudotyped with the HA of A/Wisconsin/67/2022 [H1N1]) except for r15057 of Group 3, which began losing control of SIVmac239 replication early in the vaccination regimen and was not fully immunocompetent (Figure 7). H1N1-nAb titers were considerably lower than those observed against the H5N1 and H7N9 subtypes, with ID_50_ values between 1:100 and 1:1000 in the three vaccinees exhibiting detectable H1N1 PSV neutralization activity after all three vaccination doses. H3N2 PSV serum/plasma neutralization activity increased slightly between pre- and post-vaccination timepoints in both Group 2 animals (21-fold and 5-fold for r10038 and r16003, respectively), but no increases in H3N2 PSV neutralization activity were observed in Group 1 and Group 3 animals.

### 2.6. HA-Specific B Cells Are Readily Detectable in Vaccinee PBMCs Using Fluorophore-Conjugated Recombinant HA Probes

**Figure 8:**
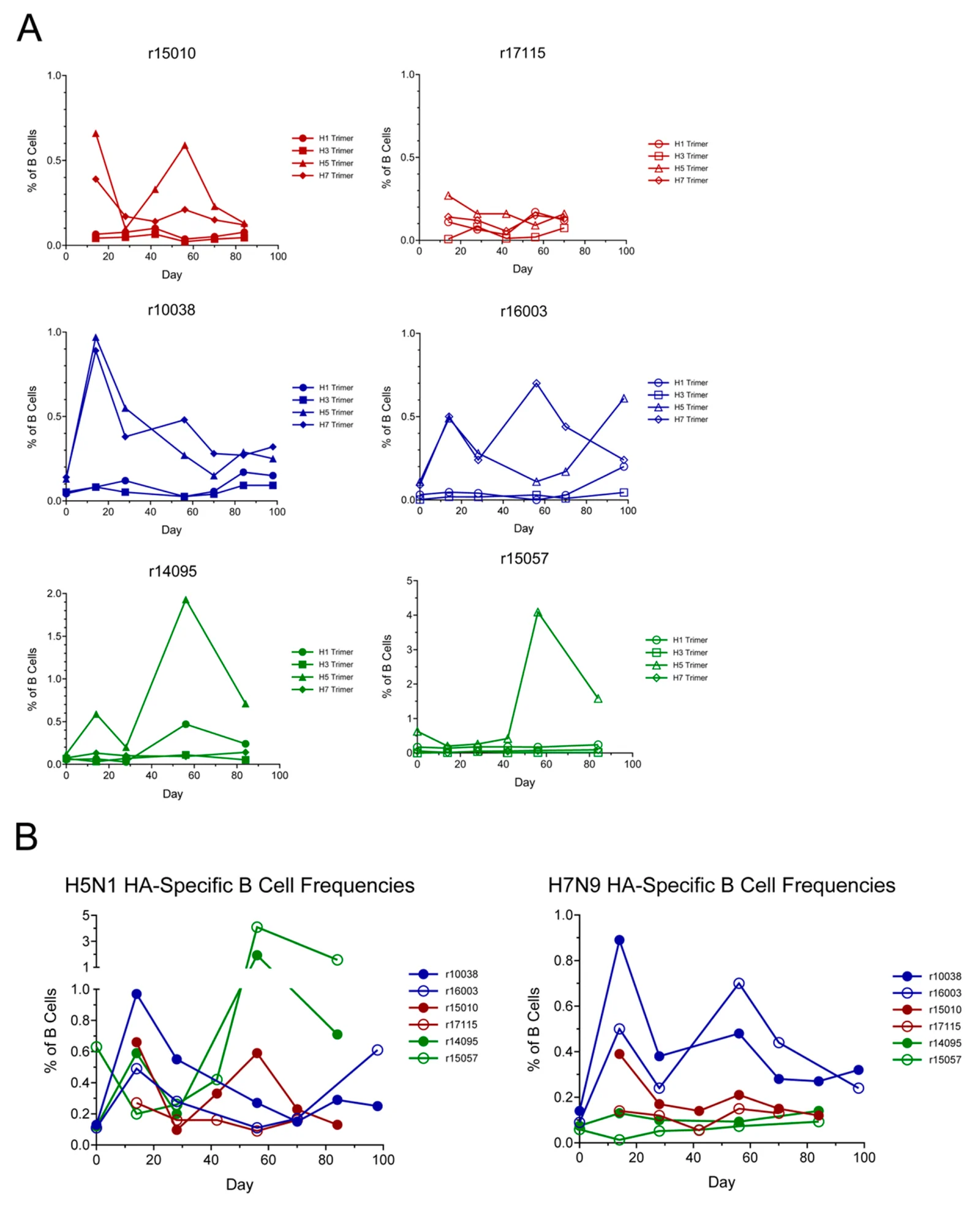
Longitudinal frequencies of HPAI HA-specific B cell populations within PBMCs of vaccinated RMs. Vaccinee PBMCs were stained with fluorophore-labeled trimeric HA probes and a cocktail containing fluorophore-conjugated mAbs and a fluorescent viability dye, then analyzed by flow cytometry. (**A**) Longitudinal frequencies of B cells specific for the HAs of the H1N1, H3N2, H5N1, and H7N9 subtypes in each vaccinated RM. (**B**) Longitudinal frequencies of HPAI HA-specific B cells in all vaccinees. Vaccinees received intramuscular injections of mRNA-LNPs and/or rAd5 vectors encoding HPAI HA stems only (Group 1, red), full-length HPAI HAs only (Group 2, blue), or H5N1 HAs only (Group 3, green). Plots depict frequencies of B cells specific for the indicated fluorophore-conjugated trimeric HA probe. Rhesus B cells were defined as live CD20^+^ HLA-DR^+^ CD3^–^ CD8a^–^ CD14^–^ CD16^–^ lymphocytes.

We performed longitudinal staining of vaccinee PBMCs to monitor frequencies of B cells specific for HPAI HAs and seasonal IAV HAs throughout the vaccination regimen. Using our trimeric biotinylated HA probes conjugated to fluorophore-labeled streptavidin, we stained vaccinee PBMCs and analyzed the longitudinal dynamics of vaccine-induced B cell populations. Populations of both H5- and H7-specific B cells were readily detectable within the PBMCs of Group 2 vaccinees, with frequencies peaking near 1% in r10038 and near 0.6% in r16003 (Figure 8). Unsurprisingly, Group 3 vaccinees receiving the H5-only vaccination regimen possessed high frequencies of H5-specific B cells but near-undetectable frequencies of H7-specific B cells. HA-specific B cell frequencies varied substantially between the two Group 1 animals; r15010 possessed moderate frequencies of H5- and H7-specific B cells, while HA-specific B cells were near-undetectable in r17115. B cells specific for the HA of the seasonal IAV H1N1 subtype became detectable in several vaccinees later in the vaccination regimen (r10038, r16003, and r14095). However, B cells specific for the HA of the seasonal IAV H3N2 subtype were largely absent in all six vaccinees throughout the duration of the vaccination regimen.

## 3. Discussion

The H5N1 panzootic of the 2020s has created an unprecedented number of opportunities for crossover infections of humans with HPAI viruses. Tens of thousands of mammalian infections have been confirmed—most notably in marine mammals and dairy cattle—and evidence of viral adaptation for mammalian replication and mammal-to-mammal transmission has been observed [3,4,5,6,7,8,9]. Historical HPAI case fatality rates have been alarmingly high (near 50%), and an HPAI epidemic or pandemic in human populations could have devastating consequences for both human health and the global economy [1,39]. It is therefore imperative that efficacious countermeasures are developed to prevent and contain the spread of HPAI viruses in humans before it is too late.

In this pilot study, we assessed the immunogenicity of several novel HPAI-tailored vaccination regimens in IAV-naïve NHPs to facilitate the development of HPAI vaccination regimens in humans and the isolation of potent and broadly reactive nmAbs for use in containing HPAI outbreaks in humans. We performed prime–boost–boost vaccination regimens in RMs using mRNA-LNPs and rAd5 vectors encoding the full-length hemagglutinin proteins or hemagglutinin stem domains of the HPAI H5N1 and H7N9 subtypes, eliciting HA-binding IgG in all six vaccinees and exceptional potent serum neutralization activity against both HPAI subtypes in two vaccinees. Animals receiving vaccines encoding only the HA stem domain did not exhibit serum HPAI neutralization activity, despite developing HA-binding IgG. All six vaccinees developed cross-reactive binding IgG specific for the HAs of the H1N1 seasonal IAV subtype and the H6N2 LPAI subtype, and five of six vaccinees developed binding IgG specific for the HAs of the H9N2 and H10N8 LPAI subtypes. However, only the two animals receiving vaccines encoding the HA stem domain alone (and no full-length HAs) developed binding IgG specific for the HA of the H3N2 seasonal IAV subtype. These results, while preliminary in nature, are illustrative of the challenges inherent in developing influenza vaccines and eliciting potent and broadly reactive nmAbs. However, we demonstrate that mRNA-LNP- and rAd5-based vaccination regimens are efficacious in eliciting potent serum HPAI neutralization activity in NHPs, providing proof-of-concept for further evaluation of such HPAI vaccination strategies.

These results provide insight into the ontogeny and cross-reactivity of humoral immune responses directed against the HA proteins of multiple IAV and HPAI subtypes in IAV-naïve primates. Importantly, we observed that, in the absence of prior antigenic exposures, primates can develop potent nAb responses against both the H5N1 and H7N9 HPAI subtypes following a prime–boost–boost vaccination regimen. Indeed, the peak serum H5N1 ID_50_ titers of ~1:100,000 and H7N9 ID_50_ titers of ~1:50,000 observed in two of our vaccines are comparable to, and may exceed, those reported in prior human [40,41,42,43,44,45,46,47,48,49,50,51,52,53,54,55,56,57,58,59] and nonhuman [60,61,62,63,64,65,66,67,68] primate HPAI vaccination trials. Previously, the highest reported H5N1 serum neutralization titers in humans were approximately 1:20,000, observed in two separate vaccination studies utilizing either a matrix M-adjuvanted virosomal H5N1 vaccine [40] or an H5 DNA prime followed by a whole-inactivated H5N1 boost [41]. Serum H7N9 neutralization titers of at least 1:1000 were observed in humans vaccinated with a heterologous prime–boost regimen of inactivated H7N9 vaccines [52]. H5N1 ID_50_ titers of 1:1000 and 1:5000 have been reported in multiple vaccination studies of various NHP species (RMs, cynomolgus macaques, and African green monkeys) [62,63,64,65,66,67], and an mRNA-LNP-based H7N9 HA vaccine elicited serum hemagglutination inhibition titers of 1:10,000 in cynomolgus macaques [60].

The high serum HPAI neutralization activity observed in the present study relative to previous vaccine trials can likely be attributed to several factors. Unlike in vaccination studies of humans that have undergone repeated heterologous IAV exposures throughout their lifetimes, our RMs were IAV-naïve prior to HPAI vaccination, likely permitting the development of potent *de novo* nAb responses optimized for the HAs of the H5N1 and H7N9 subtypes. Original antigenic sin may therefore limit the potency of HPAI-nAb responses in humans. Second, the administration of three vaccination doses in the present study, rather than the two doses used in most previous primate HPAI vaccination studies, likely facilitated the development of higher nAb titers in our vaccinees. Finally, in contrast to most previous HPAI vaccination studies, our study utilized not only heterologous HA immunogens, but also heterologous vaccination modalities. All three vaccination groups received two doses of mRNA-LNP vaccines and one dose of an adenovirus-vectored (rAd5) vaccine. Numerous studies, including those of individuals receiving various combinations of heterologous SARS-CoV-2 vaccines, have demonstrated that vaccine-induced immune responses are often superior when using heterologous vaccine modalities in prime–boost regimens rather than administering an identical vaccine multiple times [69,70,71,72,73,74,75,76].

One limitation of our vaccination study is the use of SIV-infected RMs to evaluate vaccine-induced immune responses. While the SIV-infected RMs, except r15057, were elite controllers and maintained low viremia throughout the study without developing signs or symptoms of immunodeficiency, these animals may have a higher degree of baseline inflammation than SIV-negative animals. However, our results suggest that well-controlled SIV infection did not impair the magnitude or breadth of vaccine-induced HA-specific Ab responses. Groups 1 and 2 each contained one elite controller and one SIV-negative animal, and longitudinal trajectories of HPAI HA-binding and neutralizing Ab titers were nearly identical for the animals in each of these groups. In contrast, the marked divergence in HPAI HA-binding and neutralizing Ab titers between r14095, an SIV elite controller, and r15057, a former SIV elite controller that lost viremic control early in the vaccination regimen, demonstrate both the deleterious effects of SIV-induced immunodeficiency on vaccine-induced Ab responses and the relative preservation of Ab responses to heterologous antigens in elite controllers. Previous studies have demonstrated that while HIV/SIV elite controllers can exhibit some degree of immune dysfunction and chronic inflammation, their ability to mount adaptive immune responses to heterologous pathogens often remains largely intact [77,78,79,80].

Due to the ubiquity of HA-specific B cells in humans, which are elicited and reactivated by repeated vaccination and/or infection from a very early age, refs. [81,82,83,84,85] it is difficult to predict how a similar HPAI HA-based vaccination regimen would perform in human populations. The original HA-specific B cells in humans are nearly always elicited by the seasonal H1N1 and H3N2 IAV subtypes or IBV, then restimulated by infection or vaccination with different strains of the same subtypes. Vaccination with heterologous HPAI HAs could preferentially activate seasonal IAV HA-specific memory B cells that cross-react with HPAI HAs, rather than naïve B cells. Because the HA stem is more conserved among IAV subtypes, these reactivated cross-reactive memory B cells are often specific for the HA stem [81,84,85], which is a suboptimal neutralization epitope because it is not directly involved in binding to sialic acid on host cells [86,87,88]. Indeed, we observed that our Group 1 HA stem-only vaccinees exhibited relatively high titers of HPAI HA-binding IgG but virtually no HPAI neutralization activity, suggesting that some HA stem-specific Abs lack potent neutralization capabilities [86,87,88,89,90,91] (except for a few rare mAbs) [92,93,94,95]. The HA stem also exhibits poorer immunogenicity than the HA head (or full-length HA protein), which may partially contribute to the low levels of nAbs observed in our HA stem-only vaccinees [17,18,19,20,87]. The lower immunogenicity of our HA stem-encoding vaccines may also be due to the lower levels of surface protein expression exhibited by our HA stem constructs relative to our full-length HA constructs, although the flow cytometric analyses of our vaccine constructs in Figure 1 are not truly quantitative due to potential differences in staining intensity based on the relative avidities of the HA-specific staining Abs for different HA subtypes and subdomains. In contrast, RM vaccinees receiving at least one dose of a vaccine encoding a full-length H5N1 HA developed strong H5N1 serum nAb responses (Group 2 and 3 vaccinees) along with higher levels of both H5N1 HA trimer- and HA stem-binding IgG. Group 2 vaccinees (full-length HA only, both H5N1 and H7N9) developed slightly lower levels of H7N9 HA stem-binding IgG than Group 1 vaccinees (HA stem only, both H5N1 and H7N9) and higher levels of H7N9 HA trimer-binding IgG than Group 1 vaccinees, suggesting that the potent serum H7N9 neutralization activity in Group 2 animals is largely mediated by HA head-specific Abs. Indeed, in our vaccination cohort, we observed stronger positive associations between serum HPAI neutralization activity and HPAI HA trimer-binding titers than between serum HPAI neutralization activity and HPAI HA stem-binding titers, suggesting that HA head-specific Abs may be a more important contributor to neutralization activity. However, these associations were not statistically significant, and further vaccination studies with larger numbers of animals will be required to determine whether these observations are reproducible. Additionally, although we can infer that the presence of the HA head in the context of native full-length HA is a major contributor to the enhanced neutralization titers seen in animals vaccinated with full-length HA relative to the HA stem alone, HA head-only immunogens were not utilized for vaccination or in vitro assessments of Ab specificity in this study. Therefore, the use of HA head-only immunogens in future studies could provide further insight into our preliminary finding that full-length HA immunogens tend to elicit substantially higher titers of HPAI-neutralizing Abs than HA stem antigens. Thus, despite providing proof-of-concept for an mRNA-LNP- and rAd5-based prime–boost–boost HPAI vaccination regimen to elicit high levels of HPAI-nAbs in IAV-naïve individuals, a major limitation of this study is that it does not account for the near-ubiquitous pre-existing IAV immunity in the human population. Future studies should attempt to assess the immunogenicity of similar HPAI vaccination regimens in humans and analyze the breadth and potency of subsequent nAb responses to determine whether such an approach would be viable in the context of an HPAI epidemic or pandemic.

The development of a universal influenza vaccine remains a major global health priority, aiming to elicit immune responses protective against not only seasonal IAV and IBV, but also HPAI and LPAI subtypes. Abs specific for the HA stem region have been a major focus of these efforts, due to the high degree of stem conservation among IAV and IBV subtypes [86]. However, as we discuss above, the HA stem is a poor neutralization epitope, and many HA stem-specific mAbs lack detectable neutralization activity [86,87,88,91,96]. In the present study, we assessed the ability of our HPAI HA vaccination regimen to elicit cross-reactive binding and neutralizing Ab responses against other IAV subtypes. Low levels of H1N1 HA trimer- and stem-binding IgG were detected in all six vaccinees, and low levels of H1N1 neutralization activity were only observed in animals receiving at least one dose of a vaccine encoding full-length H5N1 HA, again suggesting that the neutralization effect is largely mediated by HA head-specific Abs. Interestingly, H3N2 HA trimer- and stem-binding IgG were only detected in Group 1 (HA stem only) vaccinees, and only two of six vaccinees showed slight increases in H3N2 neutralization activity between pre- and post-vaccination timepoints. The poor H3N2 HA responses elicited by our vaccination regimen are problematic, since the H3N2 subtype accounts for most human influenza infections each year. Other studies have also shown that H3N2 neutralization coverage can be difficult to achieve without vaccinating with an H3N2 HA, such as the bivalent HA stem vaccination regimen composed of H1N1 and H10N8 HA stems reported by Moin et al. [97]. These results suggest that inclusion of an H3N2 HA in the vaccination regimen may be necessary to provide optimal coverage, likely due to the greater evolutionary distance between the H3N2 and H7N9 HAs than between the H1N1 and H5N1 HAs. Although we were unable to assess serum neutralization activity against the H6N2, H9N2, and H10N8 LPAI subtypes, five of our six vaccinees developed low but detectable titers of IgG binding the HAs of these LPAI subtypes. Thus, while our full-length HPAI vaccination regimen elicited potent serum neutralization activity against both the H5N1 and H7N9 subtypes, further IAV HA subtype cross-reactivity was quite limited, suggesting that enhanced neutralization breadth will require boosting with HAs of additional IAV subtypes.

One of the objectives of our study was to examine the HA-specific B cell repertoires of HPAI-vaccinated RMs and determine whether potent and/or broadly reactive HA-specific mAbs could be isolated from our vaccinees. Using fluorophore-labeled HA probes corresponding to HPAI subtypes (H5N1 and H7N9) and seasonal IAV subtypes (H1N1 and H3N2), we found that HA-specific B cells were readily identifiable in our vaccinees, suggesting that the mAbs produced by these B cells could be produced and screened individually for HA binding and IAV neutralization activity. The isolation and development of novel potent HPAI-specific nmAbs could greatly enhance our preparedness for HPAI epidemics and pandemics, much like SARS-CoV-2-nmAbs provided an effective means of treatment for many infected individuals during the COVID-19 pandemic. Indeed, the efficacy of mAb prophylaxis in protecting cynomolgus macaques against severe H5N1 infection was recently demonstrated [98]. HPAI-nmAb reagents possess considerable advantages over vaccination during the early stages of an epidemic or pandemic, providing near-immediate antiviral efficacy upon administration to a patient, regardless of immunocompetence, and providing utility as both a prophylactic and therapeutic agent to mitigate further transmission and reduce mortality.

In this preliminary proof-of-concept study, we assessed the immunogenicity and longitudinal humoral immune responses of three novel HPAI-tailored vaccination regimens, composed of nucleic acid-based vaccines encoding the full-length HAs and/or the HA stem domains of the H5N1 and H7N9 subtypes, in RMs. Beyond eliciting high titers of H5N1- and H7N9-nAbs in two vaccinees, our results provide insight into the ontogeny of vaccine-induced Ab responses to HPAI HAs in IAV-naïve individuals, which is difficult to model in humans, as well as the IAV HA subtype cross-reactivity profiles of HPAI-vaccinated individuals. Our results suggest that the IAV neutralization activity observed in our vaccinees (against the H5N1 and H7N9 HPAI subtypes and the H1N1 seasonal IAV subtype) is mostly mediated by HA head-specific Abs, and that many vaccine-induced HA stem-specific Abs lack potent neutralization activity. However, due to the small sample sizes of our vaccination groups, larger-scale vaccination trials and live virus challenge studies will be required to further validate our findings and demonstrate protective efficacy. Future studies should also assess vaccine-induced Ab responses in respiratory mucosal surfaces using nasopharyngeal swabs and/or bronchoalveolar lavages, which could provide more accurate information regarding the predicted protective efficacy of our vaccination regimens given the strong IAV tropism for the upper and lower respiratory tracts. It is also possible that differences in vaccine vector administration order (mRNA vaccine versus rAd5 vector) may have influenced immunogenicity and the overall magnitude and breadth of vaccine-induced Ab responses. Nevertheless, these results provide proof-of-concept for nucleic acid-based vaccination regimens in eliciting potent nAb responses against both the H5N1 and H7N9 HPAI subtypes in primates and provide a basis for the isolation and development of potent HPAI-nmAbs for use as prophylactic and therapeutic agents in humans. Collectively, the results of this study enhance our understanding of HPAI HA-specific Ab responses in primates and could facilitate the development of novel vaccination regimens and nmAb therapeutics to improve our preparedness for future HPAI epidemics and pandemics.

## 4. Materials and Methods

### 4.1. Vaccine Design and In Vitro Validation

Candidate vaccine constructs were first evaluated based on cell surface protein expression following transfection of Expi293F cells (Gibco) with DNA vectors. Codon-optimized DNA inserts encoding various membrane-bound HA proteins and subdomains were synthesized by Integrated DNA Technologies (Coralville, IA, USA), then subcloned into pcDNA3.4 using the NEBuilder HiFi DNA Assembly master mix (New England Biolabs, Ipswich, MA, USA). Following sequence confirmation via Sanger and/or next-generation sequencing methods (Azenta Life Sciences, Burlington, MA, USA), plasmids were transfected into Expi293F cells. Surface HA expression was assessed 48 h post-transfection by staining cells with HA-specific primary mAbs (MEDI8852, FI6v3, H011) or polyclonal Abs (anti-H5N1, Sino Biological, Beijing, China; anti-H7N9, Thermo Fisher Scientific, Waltham, MA, USA), followed by secondary staining with phycoerythrin-conjugated anti-IgG secondary Abs (PE anti-human IgG Fc, Jackson Immunoresearch, West Grove, PA, USA; PE anti-rabbit IgG, BioLegend, San Diego, CA, USA) and a Far Red amine-reactive viability dye (Invitrogen, Waltham, MA, USA). Human IgG and rabbit IgG isotype control mAb staining conditions were included as negative controls. Samples were acquired on a special-order LSR II cytometer (BD Biosciences, San Jose, CA, USA) and analyzed using FlowJo software v10.9 (BD Biosciences, Ashland, OR, USA). Recombinant surface HA constructs exhibiting the best expression were selected for mRNA-LNP and rAd5 vaccination vector production. mRNA-LNPs encoding the following HA proteins were produced by ProMab Biotechnologies (Richmond, CA, USA): full-length H5 (A/Chile/25945/2023 [H5N1]), full-length H7 (A/Taiwan/1/2017 [H7N9]), membrane-bound H5 stem (A/Chile/25945/2023 [H5N1]), and membrane-bound H7 stem (A/Taiwan/1/2017 [H7N9]). rAd5 vectors encoding the following HA proteins were produced by ViraQuest Inc. (North Liberty, IA, USA): full-length H5 (A/Vietnam/1203/2004 [H5N1]) and membrane-bound H5 stem (A/Chile/25945/2023 [H5N1]). mRNA-LNPs were validated in vitro by transfecting Expi293F cells (4 μg/mL final concentration) and performing the staining and flow cytometric analysis described above 48 h post-transfection. rAd5 vectors were validated by transducing Expi293F cells at multiplicity of infection (MOI) values between 10 and 10^3^ and analyzing by flow cytometry 48 h post-transfection. Samples were acquired on a special-order BD LSR II cytometer and analysis was performed using FlowJo software v10.9; a representative gating strategy is shown in Supplementary Figure S1.

### 4.2. Research Animals and Ethics Statement

Six Indian RMs (*Macaca mulatta*) were used in this study (Table 1). RMs were housed at the Wisconsin National Primate Center (WNPRC) at the University of Wisconsin-Madison and cared for in compliance with the guidelines of the Weatherall report and the National Research Council’s *Guide for the Use and Care of Laboratory Animals* [99,100]. Experimental protocols were approved by the University of Wisconsin Graduate School Animal Care and Use Committee. Detailed descriptions of animal caretaking procedures (e.g., food, housing, enrichment activities, standard medical care) can be found in the “Research animals and ethics statement” sections of the Materials and Methods sections of our previous studies performed at the WNPRC [35,101,102]. For vaccinations and blood draws, animals were anesthetized with ketamine (intramuscular, 5–12 mg/kg). Animals exhibiting signs or symptoms of SIV-induced immunodeficiency (e.g., opportunistic infections) were euthanized to minimize potential suffering by first administering general anesthesia (intramuscular ketamine, ≥15 mg/kg), then administering an intravenous overdose of sodium pentobarbital (≥50 mg/kg).

### 4.3. Animal Vaccinations

The six RMs recruited for this study were divided into three groups of two animals. mRNA-LNPs were administered as intramuscular injections to the quadriceps either individually (100 μg per dose) or as a cocktail of two mRNA-LNPs mixed at a 1:1 ratio (50 μg of each mRNA-LNP). The rAd5 vector encoding full-length H5 (A/Vietnam/1203/2004 [H5N1]) was administered as an intramuscular injection split between both quadriceps, for a final dosage of 4.8 × 10^10^ viral particles per animal. The rAd5 vector encoding the membrane-bound H5 stem (A/Chile/25945/2023 [H5N1]) was also administered as an intramuscular injection split between both quadriceps, but instead at a final dosage of 3.5 × 10^10^ viral particles per animal.

### 4.4. Sample Processing and Cryopreservation

RM PBMCs and plasma were isolated from EDTA-anticoagulated blood by density gradient centrifugation using Ficoll-Paque Plus (Cytiva, Marlborough, MA, USA). PBMCs were washed in R10 medium (RPMI 1640 with GlutaMAX or L-glutamine, 10% heat-inactivated fetal bovine serum, 1× antibiotic–antimycotic), and red blood cells were lysed by resuspending PBMCs in ACK lysis buffer (Gibco, Thermo Fisher Scientific, Waltham, MA, USA) and incubating at room temperature for 5 min. Following an additional wash in R10 medium, PBMCs were enumerated, pelleted via centrifugation, and resuspended in cold cryopreservation medium (45% RPMI 1640 with GlutaMAX or L-glutamine, 45% heat-inactivated fetal bovine serum, 10% DMSO). PBMCs were cooled to −80 °C at a controlled rate of 1 °C/min, then transferred to liquid nitrogen freezers for long-term storage. RM serum was isolated via centrifugation of blood collected in serum-separating tubes (BD). Both plasma and serum were stored at −80 °C.

### 4.5. Recombinant IAV HA Production

Codon-optimized DNA inserts encoding secreted, histidine-tagged trimeric IAV HA proteins and trimeric IAV HA stems were produced by Integrated DNA Technologies (Coralville, IA, USA). HA stem probe design was adapted from that of Corbett et al. [103]. DNA inserts were subcloned into pcDNA3.4 by Gibson assembly using NEBuilder HiFi DNA Assembly master mix (New England Biolabs, Ipswich, MA, USA). Plasmid coding DNA sequences were confirmed via Sanger and/or next-generation sequencing (Azenta Life Sciences, Burlington, MA, USA). Recombinant HAs and HA stems were produced by transient transfection of Expi293F and/or ExpiCHO cells and subsequent nickel-nitrilotriacetic acid (Ni-NTA) affinity chromatography from culture supernatant. Purified proteins were validated by SDS-PAGE with Coomassie staining (under crosslinking [dithiobis(succinimidyl propionate)], reducing [β-mercaptoethanol], and non-reducing conditions) and ELISA using IAV HA-specific mAbs MEDI8852 and FI6v3. HA monomers for the H6N2, H9N2, and H10N8 subtypes were purchased from Sino Biological.

### 4.6. IAV HA ELISAs

Titers of IAV HA-binding Abs in RM vaccinee serum were measured by ELISA. Plates were coated with 5 μg/mL HA in bicarbonate coating buffer overnight at 4 °C. The following day, plates were washed with PBS/Tween 20, then blocked with 5% skim milk in PBS for 1 h at 37 °C. Following another wash with PBS/Tween 20, plates were incubated with serial dilutions of RM vaccinee serum in 5% skim milk in PBS for 1 h at 37 °C. Plates were washed, then incubated with an HRP-conjugated goat anti-human IgG secondary Ab (Southern Biotech (Birmingham, AL, USA) 2045-05, 1:10,000 dilution in 5% skim milk in PBS) for 1 h at 37 °C. After a final wash with PBS/Tween 20, plates were developed for 2–3 min using 3,3′,5,5′-tetramethylbenzidine (TMB) substrate. TMB stop solution was added to terminate the HRP-catalyzed reaction, and absorbance values at 450 nm were acquired using a BioTek Cytation7 plate reader (Agilent Technologies, Santa Clara, CA, USA).

### 4.7. IAV PSV Production

Lentiviral PSVs corresponding to IAV strains of HPAI and non-HPAI IAV subtypes were generated using a nanoluciferase-encoding, Env- and Vpr-deficient HIV-1 backbone (pNL4-3.NanoLuc.R^–^.E^–^). Codon-optimized DNA inserts encoding the full-length HA and neuraminidase (NA) proteins of various IAV strains (Supplementary Table S1) were synthesized by Integrated DNA Technologies (Coralville, IA, USA) and subcloned into pcDNA3.4 by Gibson assembly using the NEBuilder HiFi DNA Assembly master mix (New England Biolabs, Ipswich, MA, USA). Plasmid coding DNA sequences were confirmed by Sanger and/or next-generation sequencing (Azenta Life Sciences, Burlington, MA, USA). To generate PSVs, HEK293T cells seeded at 80% confluency were transfected with an HA-encoding plasmid, an NA-encoding plasmid, and pNL4-3.NanoLuc.R^–^.E^–^ at a (1:1):2 (HA:NA):HIV ratio using the jetPRIME DNA transfection system (Polyplus, Illkirch-Graffenstaden, France). Four hours post-transfection, media was aspirated and replaced with fresh IAV infection medium (DMEM with high glucose and GlutaMAX [Gibco], 0.3% bovine serum albumin, 25 mM HEPES, 1× antibiotic-antimycotic). At 24 h post-transfection, media was again aspirated and replaced with fresh IAV infection medium. PSV-containing supernatant was harvested at 48 h post-transfection, centrifuged at 670× *g* for 10 min to pellet residual cells, and filtered (0.45 μm) prior to aliquoting and freezing at −80 °C for long-term storage. PSV infectivity was assessed by infecting MDCK and/or MDCK-SIAT1 cells at various dilutions, then lysing cells at 48 h post-infection to quantify nanoluciferase activity using the NanoGlo Luciferase Assay kit (Promega, Madison, WI, USA).

### 4.8. IAV PSV Neutralization Assays

Neutralization assays with IAV PSVs were performed using MDCK or MDCK-SIAT1 cells. Cells were seeded in white-bottom 96-well plates at 35,000 cells/well and incubated overnight at 37 °C. The following day, IAV PSV stocks were thawed from −80 °C in a 37 °C water bath, then diluted in IAV infection medium (DMEM with high glucose and GlutaMAX [Gibco], 0.3% bovine serum albumin, 25 mM HEPES, 1× antibiotic–antimycotic) containing 1–5 μg/mL TPCK-treated trypsin (Thermo Fisher Scientific). Heat-inactivated RM serum was serially diluted in IAV infection medium containing 1–5 μg/mL TPCK-treated trypsin. Diluted PSV and serum dilutions (with technical replicates for each condition) were combined at a 1:1 ratio in a separate 96-well plate, then incubated at 37 °C for 1 h. Media was then aspirated from pre-seeded MDCK or MDCK-SIAT1 cells, and PSV-serum mixtures were transferred to the cells. MDCK cells were used for H5N1 PSV neutralization assays, while MDCK-SIAT1 cells were used for H1N1, H3N2, and H7N9 PSV neutralization assays. Following 48 h of incubation at 37 °C, media was aspirated and cells were lysed using the NanoGlo Luciferase Assay kit (Promega). Nanoluciferase activity was quantified via luminescence measurements on an Agilent BioTek Cytation7 plate reader. Relative luminescence values (RLUs) for each condition were divided by the RLU value for the serum-free negative control condition, then multiplied by 100 to yield % maximal infection values. Half-maximal inhibitory dilution (ID_50_) values were calculated by performing sigmoidal-fit nonlinear regression analysis in GraphPad Prism 10 (Boston, MA, USA). Inter-assay variability was managed by including non-reactive naïve serum conditions for each assay (to assess for baseline neutralization activity and non-specific infection inhibition) and by using PSV stocks from identical batches for a given IAV subtype when possible.

### 4.9. Live H5N1 Plaque-Reduction Neutralization Tests (PRNTs)

PRNTs with live H5N1 were performed essentially as previously described [104]. Briefly, MDCK cells were seeded at 80% confluency in tissue culture-treated 96-well plates and incubated overnight at 37 °C. Serial dilutions of RM vaccinee serum were incubated with live H5N1 (A/dairy cattle/Texas/24008749001/2024, generated via reverse genetics) [12] for 1 h at 37 °C, then added to pre-seeded MDCK cells. After an additional 1 h of incubation at 37 °C, cells were overlaid with media containing 0.5% agarose. Following 72 h of incubation at 37 °C, cells were fixed with formaldehyde for 2 h, then stained with 0.25% crystal violet to visualize plaques. Plaques were enumerated manually using a backlight plate reader. PRNT_50_ values were defined as the lowest serum dilutions preventing plaque formation in at least 50% of wells across technical replicates.

### 4.10. Flow Cytometry Analysis of IAV HA-Specific B Cells

IAV HA-specific B cells were detected by staining vaccinee PBMCs with fluorophore-labeled HA trimer probes. Biotinylated HA trimer probes (containing an AviTag and a histidine tag at the C-terminus) were produced by transient transfection of Expi293F cells with plasmids encoding the HA trimer and *E. coli* BirA at a 4:1 ratio, as previously described [105]. Probes were purified from culture supernatant by Ni-NTA affinity chromatography and validated by SDS-PAGE with Coomassie staining under various conditions (as described above) and by sandwich ELISA to confirm biotinylation. Sandwich ELISA was performed by coating plates overnight with IAV HA-specific mAbs MEDI8852 or FI6v3, incubating with serial dilutions of purified, *birA* co-transfected HA trimers, and detecting biotinylated trimers using HRP-conjugated streptavidin (Southern Biotech). Biotinylated HA trimer probes were conjugated to fluorophore-labeled streptavidins (APC, BV421, BV785, or PE; BioLegend), then used to stain vaccinee PBMCs for 30 min at room temperature. Following a wash in FACS buffer (1% heat-inactivated FBS in PBS), PBMCs were stained with the following cocktail of fluorophore-conjugated mAbs and fluorescent amine-reactive viability dye for 30 min at room temperature to permit identification of rhesus B cells: anti-CD20 PE-Cy7 (clone 2H7, BioLegend), anti-HLA-DR BV605 (clone L243, BioLegend), anti-CD27 FITC (clone O323, BioLegend), anti-CD3 PerCP-Cy5.5 (clone SP34-2, BD Biosciences), anti-CD8a BV510 (clone RPA-T8, BioLegend), anti-CD14 BV510 (clone M5E2, BioLegend), anti-CD16 BV510 (clone 3G8, BioLegend), aqua amine-reactive viability dye (Invitrogen). Cells were washed with FACS buffer, fixed with BD Cytofix (containing 4.2% formaldehyde) for 20 min at 4 °C, and washed once more prior to acquisition on a special-order BD LSR II flow analyzer. Rhesus B cells were defined as live CD20^+^ HLA-DR^+^ CD3^–^ CD8a^–^ CD14^–^ CD16^–^ lymphocytes. Analysis was performed using FlowJo software v10.9; gating strategy is shown in Supplementary Figure S2. Frequencies of HA trimer^+^ B lymphocytes at post-vaccination timepoints were compared with baseline pre-vaccination (day 0) frequencies for each animal to assess background/baseline reactivity to each HA trimer probe and fluorophore-conjugated streptavidin.

## Figures and Tables

**Table 1:** Animal characteristics.

Vaccination Group	Animal ID	MHC Class I Alleles ^a^	Age (Years) ^b^	Weight (kg) ^c^	Sex	SIVmac239 Infection Status
1—HA Stem Only	r15010	Mamu-A*02, Mamu-B*17	9.2	15.1	M	SIV− (challenged)
	r17115	Mamu-B*08	6.4	10.2	M	SIV+ elite controller ^d^
2—Full-Length HA Only	r10038	Mamu-B*08	13.9	9.8	F	SIV− (challenged)
	r16003	Mamu-B*08	8.2	5.7	F	SIV+ elite controller
3—H5N1 HA Only	r14095	Mamu-B*08	9.5	8.8	F	SIV+ elite controller
	r15057	Mamu-B*08	8.5	7.9	M	SIV+ elite controller ^e^

^a^ RM MHC class I alleles relevant for spontaneous control of SIVmac239 viremia and/or characterization of SIV-specific CD8+ cytotoxic T lymphocyte populations. ^b^ Age at time of first IAV HA vaccination. ^c^ Weight at time of first IAV HA vaccination. ^d^ We defined elite control of SIVmac239 viremia as plasma viral loads ≤10,000 vRNA copies/mL for ≥4 weeks during subacute/chronic infection. ^e^ r15057 initially exhibited low viremia characteristic of an SIV elite controller, but lost control of viremia early in the vaccination regimen.

**Table 2:** Vaccinee serum neutralization activity against live HPAI viruses.

Vaccination Group	Animal ID	H5N1 PRNT_50_^a^ (A/Dairy Cattle/Texas/24008749001/2024)	
		Pre-Vaccination	Post-Vaccination ^b^
1—HA Stem Only	r15010	128	128
	r17115	1024	128
2—Full-Length HA Only	r10038	256	131,072
	r16003	64	≥4096
3—H5N1 HA Only	r14095	512	≥4096
	r15057	512	2048

^a^ PRNT_50_ is reported as the reciprocal serum dilution preventing plaque formation in at least 50% of wells across technical replicates. ^b^ Vaccinee serum sample from two weeks following the third vaccination dose (day 84 for Groups 1 and 2; day 70 for Group 3).

**Table 3:** Correlations between HPAI neutralization titers and titers of HA trimer- and stem-specific Abs in HPAI-vaccinated RMs four weeks after the third vaccination dose.

Immunological Parameter 1	Immunological Parameter 2	n ^a^	Correlation Coefficient (Spearman r)	*p*-Value
H5N1 PSV Reciprocal ID_50_	H5N1 HA Trimer AUC	5	0.8721	0.1000
	H5N1 HA Stem AUC	5	0.5643	0.4000
H7N9 PSV Reciprocal ID_50_	H7N9 HA Trimer AUC	4	0.8000	0.3333
	H7N9 HA Stem AUC	4	−0.8000	0.3333

^a^ For H5N1 correlation analyses, all immunocompetent animals receiving at least one dose of an H5N1 HA-encoding vaccine were included (Groups 1 and 2, and r14095 of Group 3). For H7N9 correlation analyses, only animals receiving at least one dose of an H7N9 HA-encoding vaccine were included (Groups 1 and 2).

## Data Availability

Data are available upon reasonable request from the authors.

## Supplementary Materials

The following supporting information can be downloaded at https://www.mdpi.com/article/10.3390/v18090981/s1.
