# Supplementary material for: Elicitation of Potent H5N1- and H7N9-Neutralizing Antibody Responses in Rhesus Macaques by Sequential Heterologous Vaccination with mRNA and Adenoviral Vectors Encoding Full-Length Hemagglutinins
Source: Viruses. 2026 Sep 7;18(9):981. doi: 10.3390/v18090981 (PMC13611895; doi:10.3390/v18090981)
Supplement: Supplementary file 1 [file viruses-18-00981-s001.zip › viruses-4410096-supplementary.pdf]

Supplementary Material

1 Supplementary Figures and Tables

Supplementary Table S1. IAV strain information.

Reagent	Hemagglutinin (HA) Origin	Neuraminidase (NA) Origin
H5N1 TX24 (live virus)	A/dairy cattle/Texas/24008749001/2024 (H5N1)	A/dairy cattle/Texas/24008749001/2024 (H5N1)
mRNA-LNP H5 CL23 FL	A/Chile/25945/2023 (H5N1)	None
mRNA-LNP H7 TW17 FL	A/Taiwan/1/2017 (H7N9)	None
mRNA-LNP H5 CL23 Stem	A/Chile/25945/2023 (H5N1)	None
mRNA-LNP H7 TW17 Stem	A/Taiwan/1/2017 (H7N9)	None
rAd5 H5 VN04 FL	A/Vietnam/1203/2004 (H5N1)	None
rAd5 H5 CL23 Stem	A/Chile/25945/2023 (H5N1)	None
H1N1 WI22 PSV	A/Wisconsin/67/2022 (H1N1)	A/Vietnam/1203/2004 (H5N1)
H3N1 DW21 PSV	A/Darwin/9/2021 (H3N2)	A/Vietnam/1203/2004 (H5N1)
H5N1 CL23 PSV	A/Chile/25945/2023 (H5N1)	A/Vietnam/1203/2004 (H5N1)
H7N9 AN13 PSV	A/Anhui/1/2013 (H7N9)	A/Anhui/1/2013 (H7N9)
H1 WI19 Trimer	A/Wisconsin/588/2019 (H1N1)	None
H1 WI22 Trimer	A/Wisconsin/67/2022 (H1N1)	None
H3 BK79 Trimer	A/Bangkok/1/1979 (H3N2)	None
H3 MA22 Trimer	A/Massachusetts/18/2022 (H3N2)	None
H5 CL23 Trimer	A/Chile/25945/2023 (H5N1)	None
H7 TW17 Trimer	A/Taiwan/1/2017 (H7N9)	None
H6 CG10 Monomer	A/chicken/Guangdong/C273/2011 (H6N2)	None
H9 HK09 Monomer	A/Hong Kong/35820/2011 (H9N2)	None
H10 JD13 Monomer	A/Jiangxi-Donghu/346/2013 (H10N8)	None
H1 WI22 Stem	A/Wisconsin/67/2022 (H1N1)	None
H3 DW21 Stem	A/Darwin/9/2021 (H3N2)	None
H5 CL23 Stem	A/Chile/25945/2023 (H5N1)	None
H7 TW17 Stem	A/Taiwan/1/2017 (H7N9)	None

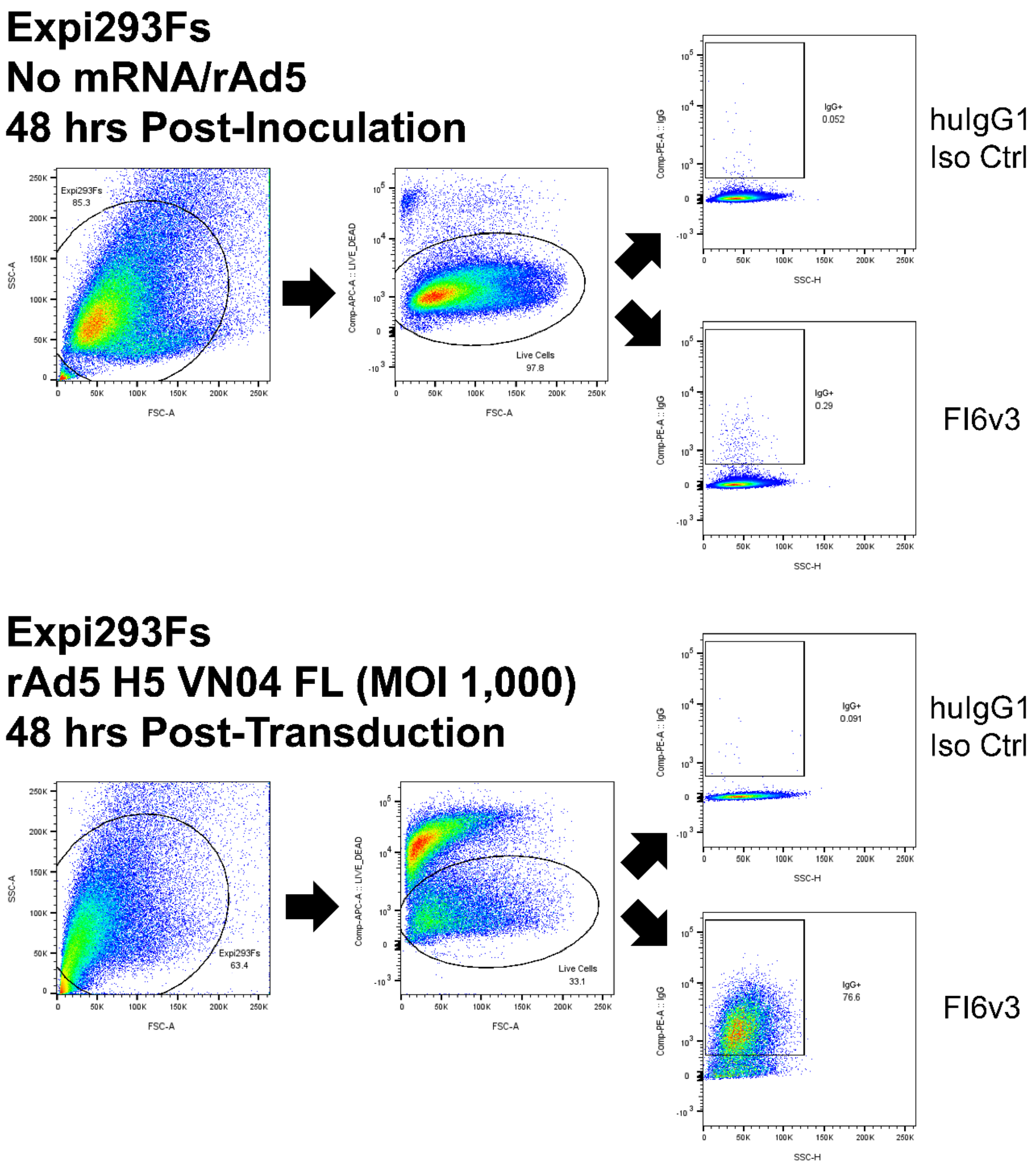

Supplementary Figure S1. Gating strategy for flow cytometric analysis of IAV HA expression from mRNA-LNPs and rAd5 vectors in Expi293F cells. Representative flow cytometry plots of Expi293Fs after 48 hours of culture, without mRNA-LNP transfection or rAd5 transduction (top), or following transduction with a rAd5 vector encoding the full-length HA protein of A/Vietnam/1203/2004 (H5N1) at an MOI of 1,000 (bottom). Cells were first stained with the indicated primary mAb (a SARS-CoV-2 RBD-specific huIgG1 serving as an isotype control, or the IAV bnmAb FI6v3), then with a Far Red amine-reactive viability dye and a PE-conjugated anti-human IgG secondary Ab.

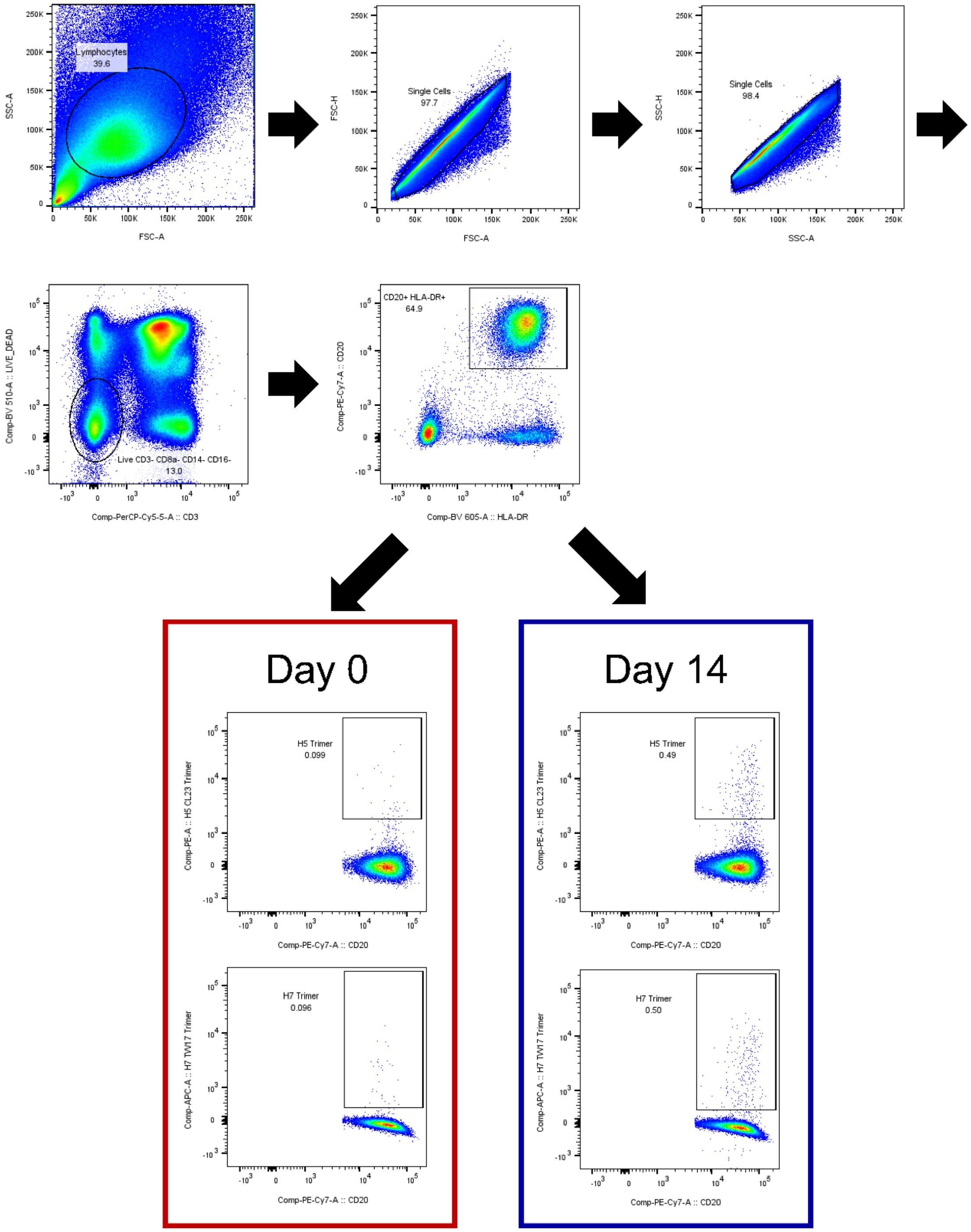

Supplementary Figure S2. Gating strategy for flow cytometric analysis of IAV HA-specific B cells in vaccinee PBMCs. Rhesus B cells were defined as live CD20^{+} HLA-DR^{+} CD3^{-} CD8a^{-} CD14^{-} CD16^{-} lymphocytes. Plots depict representative data from r16003, comparing frequencies of H5 and H7 trimer-specific B cells at the day 0 (pre-vaccination) and day 14 timepoints.
